# Lactucopicrin promotes fatty acid β‐oxidation and attenuates lipid accumulation through adenosine monophosphate‐activated protein kinase activation in free fatty acid‐induced human hepatoblastoma cancer cells

**DOI:** 10.1002/fsn3.4176

**Published:** 2024-04-18

**Authors:** Huiwen Tan, Na Mi, Fenglian Tong, Rui Zhang, Adalaiti Abudurexiti, Yi Lei, Yewei Zhong, Junlin Yan, Jian Yang, Xiaoli Ma

**Affiliations:** ^1^ College of Pharmacy Xinjiang Medical University Urumqi Xinjiang China; ^2^ Affiliated Hospital of Chongqing Three Gorges Medical College Chongqing China; ^3^ The First Affiliated Hospital of Xinjiang Medical University Urumqi Xinjiang China

**Keywords:** AMPK signaling pathway, fatty acid β‐oxidation, HepG2 cells, Lactucopicrin, non‐alcoholic fatty liver disease

## Abstract

With its annually increasing prevalence, non‐alcoholic fatty liver disease (NAFLD) has become a serious threat to people's life and health. After a preliminary research, we found that Lactucopicrin has pharmacological effects, such as lowering blood lipids and protecting the liver. Further research showed its significant activation for fatty acid β‐oxidase hydroxyacyl‐coenzyme A (CoA) dehydrogenase trifunctional multienzyme complex subunit alpha (HADHA), so we hypothesized that Lactucopicrin could ameliorate lipid accumulation in hepatocytes by promoting fatty acid β‐oxidation. In this study, free fatty acid (FFA)‐induced human hepatoblastoma cancer cells (HepG2) were used to establish an in vitro NAFLD model to investigate the molecular basis of Lactucopicrin in regulating lipid metabolism. Staining with Oil red O and measurements of triglyceride (TG) content, fatty acid β‐oxidase (FaβO) activity, reactive oxygen species (ROS) content, mitochondrial membrane potential, and adenosine triphosphate (ATP) content were used to assess the extent to which Lactucopicrin ameliorates lipid accumulation and promotes fatty acid β‐oxidation. Quantitative real‐time polymerase chain reaction (qRT‐PCR) and Western blot methods were used to explore the regulatory effects of Lactucopicrin on factors related to fatty acid β‐oxidation. Results showed that Lactucopicrin downregulated phosphorylated mammalian target of rapamycin (P‐mTOR) by activating the adenosine monophosphate‐activated protein kinase (AMPK) pathway and upregulated the messenger RNA (mRNA) and protein expression levels of coactivators (peroxisome proliferator‐activated receptor gamma coactivator 1‐alpha (PGC1α)), transcription factors (peroxisome proliferator‐activated receptor α (PPARα) and peroxisome proliferator‐activated receptor γ (PPARγ)), and oxidative factors (carnitine palmitoyltransferase 1A (CPT1A) and HADHA). This phenomenon resulted in a significant increase in FaβO activity, ATP content, and JC‐1 and a significant decrease in ROS level, TG content, and intracellular lipid droplets. With the addition of Dorsomorphin, all the effects of Lactucopicrin intervention were suppressed. In summary, Lactucopicrin promotes fatty acid β‐oxidation by activating the AMPK pathway, thereby ameliorating FFA‐induced intracellular lipid accumulation in HepG2 cells.

## Graphical abstract

Non‐alcoholic fatty liver disease (NAFLD) is a major chronic disease in contemporary society, and its immediate cause is a disorder of lipid metabolism caused by the accumulation of lipids. The alleviating effect of Lactucopicrin on intracellular lipid accumulation was achieved by promoting fatty acid β‐oxidation, which was confirmed by the upregulation of fatty acid β‐oxidation‐related factors. The adenosine monophosphate‐activated protein kinase (AMPK) pathway plays an important role in the mitigation of lipid accumulation by Lactucopicrin.

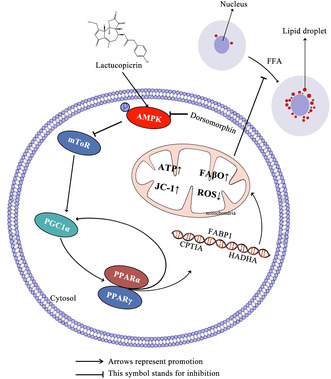

## 1 INTRODUCTION

Owing to the gradual improvement of people's living standards, a significant increase has been observed in people's high‐carbon and high‐fat intake and non‐alcoholic fatty liver disease (NAFLD) incidence (Llovet et al., 2023). According to epidemiological statistics and studies, the global prevalence of NAFLD is as high as 25%, with China having the highest prevalence and the fastest growing number of patients in the world (Powell et al., 2021; Zhou et al., 2020). However, the pathogenesis of NAFLD is complex, lifestyle intervention is still the main treatment modality for NAFLD, and drugs with clear targets and significant efficacy are not yet available on the market; therefore, effective treatments for NAFLD are urgently needed (Katsiki et al., 2022; Rong et al., 2022). The protective effect of natural plant active ingredients on NAFLD has become prominent. Therefore, the pathogenesis of NAFLD and the development of naturally active small molecules targeting the liver to block lipid accumulation are the present research hotspots for the prevention and treatment of NAFLD (Guo et al., 2022; Tarantino et al., 2021; Yao & Liu, 2022; Zhao et al., 2022).

Hairy chicory (*Cichorium glandulosum* Boiss. et Huet) from Xinjiang, China has shown good lipid‐lowering effects in traditional medicine (Yang et al., 2017). Our previous study found that chicory sesquiterpenes significantly ameliorate intrahepatic fat deposition and reduced total cholesterol and triglyceride (TG) levels in db/db mice, showing the ability to regulate lipid metabolism (Yan et al., 2022). As a common sesquiterpene constituent in Xinjiang hairy chicory, Lactucopicrin aroused our great interest. He et al. (2021) found that Lactucopicrin inhibits nuclear factor‐kappa B (NF‐κB) activation, attenuates atherosclerosis, reduces inflammation within atherosclerotic plaques, and ameliorates systemic inflammation. Weng Hui showed that (Weng et al., 2021) Lactucopicrin ameliorates sepsis by inhibiting NF‐κB activation through the down‐regulation of importin α3. Ramu Venkatesan reported that (Venkatesan et al., 2017) Lactucopicrin can enhance neurotrophic regeneration and neurotrophic effects by modulating the calcium^2+^ (Ca^2+^)/calmodulin‐dependent protein kinase II (caMKII)/(Activates transcription factor 1) ATF1 signaling pathway. Our previous study found that Lactucopicrin can improve oxidative stress imbalance and fatty degeneration; however, further study revealed that it has no significant effect on the expression of lipid synthesis‐related proteins. We then considered that Lactucopicrin might act by promoting lipolysis. Further transcriptome sequencing studies revealed that Lactucopicrin has a significant activating effect on hydroxyacyl‐coenzyme A (CoA) dehydrogenase trifunctional multifunctional enzyme complex subunit α (HADHA; Aibaidula et al., 2022), suggesting its lipid‐lowering role. However, the specific mechanism by which Lactucopicrin regulates lipid metabolism remains unclear and requires further investigation.

As a recognized fatty acid β‐oxidase, HADHA regulates fatty acid β‐oxidation (Pan et al., 2022). Given that it can activate HADHA, we hypothesized that Lactucopicrin may regulate lipid metabolism by triggering fatty acid β‐oxidation. Fatty acid β‐oxidation has also received extensive attention from scientists worldwide as a key pathway for hepatic triglyceride reduction (Selen et al., 2021). Carnitine palmitoyltransferase 1 (CPT1) is the rate‐limiting enzyme for fatty acid β‐oxidation and is usually specifically expressed in liver tissues such as carnitine palmitoyltransferase 1A (CPT1A) (Gao et al., 2011). Prior to oxidative catabolism, free fatty acids (FFAs) must be converted into activated esteryl‐coenzyme A, which is translocated by carnitine palmitoyltransferase and participates in fatty acid β‐oxidation (Mun et al., 2019). Peroxisome proliferator‐activated receptors (PPARs) have three isoforms, of which the most studied are peroxisome proliferator‐activated receptor α (PPARα) and peroxisome proliferator‐activated receptor γ (PPARγ) and peroxisome proliferator‐activated receptor β (PPARβ) has been less studied (Montaigne et al., 2021). PPARα and PPARγ are important transcriptional regulators in fatty acid β‐oxidation that can modulate the downstream target proteins involved in this process, such as CPT1A, and reduce intracellular lipid accumulation (Chu et al., 2022; Xiao et al., 2021). Peroxisome proliferator‐activated receptor γ coactivator 1α (PGC1α) acts as a coactivator that synergistically activates peroxisome proliferator‐activated receptor and is also one of the key downstream targets of the AMPK pathway (Wang et al., 2022).

Many natural plant components activate the key target adenosine monophosphate‐activated protein kinase (AMPK) (Sheng et al., 2019). AMPK, a recently discovered energy sensor, is a key enzyme protein that regulates cellular metabolism, modulates obesity, and influences lipid metabolism homeostasis (Xu et al., 2018; Zhang et al., 2020). AMPK activation significantly promotes intracellular fatty acid β‐oxidation, thereby increasing lipid metabolism, which has a positive therapeutic effect on NAFLD (Weng et al., 2023). AMPK activation also enhances PPARα expression by triggering the AMPK/PGC1α signaling axis, promoting fatty acid oxidation, and ameliorating hepatic steatosis to upregulate the expression of carnitine palmitoyltransferase 1A (CPT1A) and acyl coenzyme a oxidase 1 (ACO1) (Li et al., 2022; Lin et al., 2021). Enhanced AMPK phosphorylation also increases the rate of triglyceride metabolism and decreases lipid accumulation, showing that the AMPK pathway plays an extremely important role in lipid metabolism and significantly inhibits the onset and development of NAFLD. Asier González stated that (González et al., 2020) once activated, the catabolic regulator AMPK inevitably suppresses the mTOR activity. mTOR and AMPK have opposite functions: mTOR inhibits catabolism, activates anabolism, is regulated by AMPK, and controls multiple AMPK direct or indirect targets (Herzig & Shaw, 2018). Therefore, mTOR is a potential therapeutic target for the treatment of NAFLD and has received extensive attention from researchers worldwide. Yiguo Wang at the School of Life Sciences, Tsinghua University found that (Han et al., 2015) mTOR overactivation enhanced lipid synthesis in a model of obese and diabetic mice. Therefore, AMPK activation and mTOR inhibition can effectively treat related diseases caused by excessive lipid accumulation, such as NAFLD.

On the basis of the above studies, we hypothesized that Lactucopicrin may regulate the lipid metabolism of hepatocytes and reduce the accumulation of fat in hepatocytes by modulating the AMPK signaling pathway and its downstream target proteins, making it an ideal botanical compound for the prevention and treatment of NAFLD. In this study, 400 μM FFA was used to treat HepG2 cells to establish a model of hepatocellular steatosis. Classical molecular biology methods were used to study the molecular mechanism of Lactucopicrin to improve hepatic steatosis. This work aims to provide experimental bases for the prevention and treatment of NAFLD and the research and development of effective drugs and scientific bases for the development of sesquiterpene‐rich drug resources.

## 2 MATERIALS AND METHODS

### 2.1 Materials

The HepG2 cells were purchased from Wuhan Punosai Life Science and Technology Co. Ltd. Lactucopicrin was donated by the Chinese Academy of Sciences’ Xinjiang Institute of Physical and Chemical Sciences (purity: 98%). Dulbecco's modified Eagle medium (DMEM), phosphate‐buffered saline (PBS), and penicillin–streptomycin double antibody solution were purchased from HyClone (Thermo Fisher Scientific, USA). GlutaMAX was purchased from Gibco (Thermo Fisher Scientific). Fetal bovine serum (FBS) was purchased from BI, Israel. Oleic acid (OA) and palmitic acid (PA) were purchased from Sigma‐Aldrich. PrimeScript RT Reagent Kit with gDNA Eraser and TB Green Premix Ex Taq II (Quantitative real‐time polymerase chain reaction (PCR)) were purchased from TAKARA Japan. Human Fatty Acid β Oxidase (FAβO) ELISA Kit was purchased from Shanghai Youxuan Biotechnology Co., Ltd. Fatty‐acid‐free bovine serum albumin (BSA) was purchased from Kingmorn Biologicals, Shanghai, China. TRizol Reagent was purchased from Ambion, USA. The Cell Counting Kit‐8 (CCK8 Kit) was purchased from Shanghai Dongren. Tissue cell triglyceride (TG) content enzymatic assay kit was purchased from Beijing Pulilai. Reactive oxygen assay kit, Hoechst 33342 Staining Solution (1 mg/mL), and Oil red O staining solution kit were purchased from Solebro. The ATP assay kit and mitochondrial membrane potential assay kit (JC‐1) were purchased from Shanghai Biyuntian Biotechnology Co., Ltd. The AMPK‐specific inhibitor (Dorsomorphin) was purchased from MedChemExpress. The primers PGC1α, CPT1A, PPARα, PPARγ, HADHA, and glyceraldehyde‐3‐phosphate dehydrogenase (GAPDH) were purchased from Shanghai Biotech. The antibody HADHA was purchased from Abcam. The antibodies, such as PGC1α, phospho‐adenosine monophosphate (AMP)‐activated protein kinase α (P‐AMPKα), and adenosine monophosphate (AMP)‐activated protein kinase α (AMPKα), were purchased from Cell Signaling. The antibody GAPDH was purchased from Affinity. Antibodies, such as CPT1A, PPARα, PPARγ, P‐mTOR, mTOR, and mouse Immunoglobulin G kappa‐binding protein conjugated to horseradish peroxidase (m‐IgGκBP‐HRP), were purchased from Santa Cruz. Antibodies goat anti‐rabbit Immunoglobulin G (IgG), and goat anti‐mouse Immunoglobulin G1 (IgG1) were purchased from Southern Biotech.

### 2.2 Cell culture and handling

The HepG2 cells were cultured at 37°C with 5% CO_2_ humidity, using a medium containing 10% fetal bovine serum (FBS), a 1% mixture of penicillin–streptomycin, and 1% GlutaMAX. Oleic acid (OA) and palmitic acid (PA) were dissolved in DMEM containing 1% BSA at a ratio of 1:2 to generate FFAs with a final concentration of 400 μM. Dorsomorphin, an AMPK‐specific inhibitor, was used as a pretreatment for cells for 2 h, followed by cotreatment with Lactucopicrin (20 μM) and FFAs (400 μM) for 48 h.

### 2.3 Cell viability assay

The HepG2 cells were seeded at a density of 1 × 10^4^ cells per well in 96‐well plates and incubated overnight for 12 h. Afterward, the cells were cultured for an additional 48 h following the addition of various concentrations of Lactucopicrin. Following the Lactucopicrin treatment, 200 μL of CCK8 solution (10%) was added to each well, and the 96‐well plate was then returned to the 37°C incubator for 2 h. Subsequently, the absorbance at 450 nm was measured using an enzyme‐linked immunosorbent assay (ELISA), and the cellular viability at each concentration was determined using the provided kit's formula and statistically analyzed.

### 2.4 Determination of TG content

After washing the cells with PBS, cell lysate was added and the lysate process was completed. A portion of the lysate was then extracted and heated at 70°C for 10 min. After that, it was centrifuged at 2000 rpm for 5 min to obtain samples from each group. The remaining lysate was quantified using the bicinchoninic acid (BCA) protein assay. The triglyceride content of each cell group was determined according to the kit instructions and corrected based on the protein concentration per milligram.

### 2.5 Oil red O staining

After the cells were washed with PBS, 4% paraformaldehyde was applied to fix them. Once the fixation process was complete, the fixative was removed by rinsing with PBS. Subsequently, an Oil red O staining solution was prepared, filtered through filter paper, and placed in a water bath at 37°C for later use. The fixed cells were then incubated with freshly prepared Oil red O staining solution for 20 min, followed by an isopropanol rinse. They were then further stained with hematoxylin dye for 3 min, washed with distilled water until there was no excess staining solution, observed and photographed using a microscope, and the Oil red positive area of each group of cells in the obtained images was analyzed using ImageJ.

### 2.6 Determination of ATP content

After lysing the cells completely with cell lysis solution in an ice bath, centrifuge the sample at 12,000 ***g*** to obtain the reserved supernatant. The ATP content of each cell group was determined by following the instructions of the ATP kit to extract a portion of the supernatant, and the remainder was saved for BCA protein quantification. Before statistical analysis, the measured ATP content was corrected by protein quantification.

### 2.7 Determination of fatty acid β‐oxidase activity

Take the cell supernatant, add cell supernatant, sample dilution, and horseradish peroxidase‐labeled antibody according to the instructions of the fatty acid β‐oxidase (FaβO) kit, and incubate at 37°C for 1 h. Wash the plate five times repeatedly, add substrate A and substrate B in turn, and incubate at 37°C for 15 min with protection from light. After the incubation, add the termination solution and use a microplate reader to measure the absorbance at 450 nm. Use the linear regression curve of the standards to obtain the concentration value for each sample.

### 2.8 Measurement of reactive oxygen content

The cells in the positive control group were initially stimulated with Rosup (positive control working solution) and then incubated for 20 min at 37°C. Subsequently, the fluorescent probes were introduced through an additional 30‐min incubation at 37°C, following the guidelines provided in the ROS and Hoechst kits. After this incubation process, any excess fluorescent probes were removed using serum‐free medium. A laser confocal microscope was utilized to observe and capture images, which were then analyzed using ImageJ software to measure the fluorescence intensity within each cell population.

### 2.9 Mitochondrial membrane potential measurement

The cells in the positive control group were initially stimulated with carbonyl cyanide m‐chlorophenylhydrazone (CCCP; positive control working solution) and incubated at 37°C for 20 min. According to the instructions provided with the JC‐1 and Hoechst kit, Hoechst 33342 and the JC‐1 staining working solution were added and co‐incubated for 20 min. After incubation, the excess staining solution was removed by washing with JC‐1 staining buffer. Subsequently, DMEM was added, and each set of cells was observed using a laser confocal microscope, photographed, and the resulting images were analyzed for red–green fluorescence intensity using ImageJ.

### 2.10 Quantitative real‐time PCR

Total RNA was isolated from HepG2 cells using TRizol, and its concentration was determined with NanoDrop. The extracted RNA was then reverse transcribed using the PrimeScript RT Reagent Kit with gDNA Eraser kit from TAKARA to generate complementary DNA (cDNA). The expression levels of PPARα, PPARγ, PGC1α, CPT1A, and HADHA were quantified using quantitative real‐time PCR and TB Green Premix Ex Taq kit from TAKARA, with GAPDH serving as an internal reference for relative quantification based on the 2^−△△CT^ method. The primer pairs used in this study are listed in Table 1.

**TABLE 1:** Primers for real‐time quantitative PCR.

Gene	Sequencing	Length
CPT1A	Forward: 5′‐ACAACAAAAGCCCCTGACTG‐3′	20
	Reverse: 5′‐AGGGCAGAGAGAGCTACATCC‐3′	21
PGC1α	Forward: 5′‐TCTGAGTCTGTATGGAGTGACAT‐3′	23
	Reverse: 5′‐CCAAGTCGTTCACATCTAGTTCA‐3′	23
HADHA	Forward: 5′‐ATATGCCGCAATTTTACAGGGT‐3′	22
	Reverse: 5′‐ACCTGCAATAAAGCAGCCTGG‐3′	21
PPARα	Forward: 5′‐CCGCAATGGACCATGTAAC‐3′	19
	Reverse: 5′‐CAGCTCTAGCATGGCCTTTT‐3′	20
PPARγ	Forward: 5′‐GGGATCAGCTCCGTGGATCT‐3′	20
	Reverse: 5′‐TGCACTTTGGTACTCTTGAAGTT‐3′	23
GAPDH	Forward: 5′‐CAGGAGGCATTGCTGATGAT‐3′	20
	Reverse: 5′‐GAAGGCTGGGGCTCATTT‐3′	18

### 2.11 Western blot

After rinsing the cell surface with PBS, the cells were lysed using a cell lysis solution containing protease inhibitor and phosphatase inhibitor, followed by the extraction of total protein. The protein samples were then separated by electrophoresis and transferred to polyvinylidene difluoride (PVDF) membranes. Subsequently, they were treated with 5% skim milk for 1 h and then underwent overnight incubation with the primary antibody. Afterward, the membranes were washed four times with PBST (i.e. PBS supplemented with Tween‐20; 10 min each time) and then incubated with the corresponding secondary antibodies for 1 h, followed by the same washing process. Electroluminescence (ECL) solution was then carefully added, and the film was developed in the darkroom before being scanned to obtain images for subsequent analysis and processing using ImageJ.

### 2.12 Statistical analysis

All data were expressed as mean ± standard deviation, and one‐way analysis of variance (ANOVA) was used for comparison between multiple groups. *p < .05, **p < .01, ***p < .001, ^****^ *p* < .0001, ^ns^ *p* > .05 (not significant, ns).

## 3 RESULTS

### 3.1 Lactucopicrin attenuates FFA‐induced lipid accumulation in HepG2 cells

**FIGURE 1:**
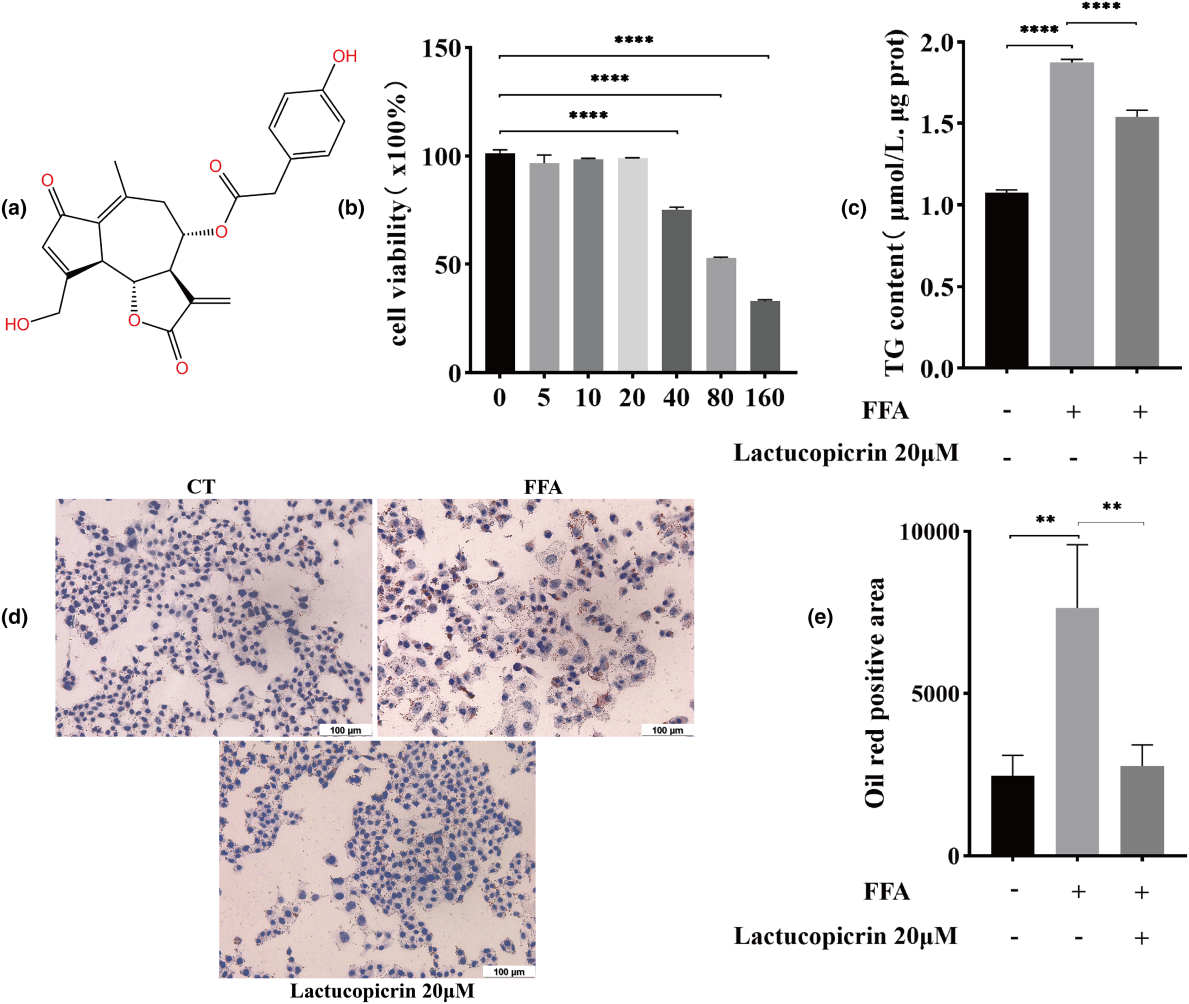
Lactucopicrin reduces FFA‐induced lipid accumulation in HepG2 cells. (a) The chemical structure of Lactucopicrin. (b) Cytotoxicity of Lactucopicrin was measured by the CCK8 method. (c) Measurement of intracellular TG content. (d, e) Lipid accumulation was determined via Oil red O staining. Scale bars, 100 μm. Control (CT), FFA 400 μM (FFA), FFA 400 μM + Lactucopicrin 20 μM (Lactucopicrin 20 μM). Data were expressed as mean ± SD for three separate experiments, **p < .01, ****p < .0001.

The CCK8 method was used to determine the safe dose of Lactucopicrin, and cells were exposed to varying concentrations of 0, 5, 10, 20, 40, 80, and 160 μM Lactucopicrin for 48 h. The objective was to assess Lactucopicrin's cytotoxic impact and determine the most effective concentration for HepG2 cell experiments. Lactucopicrin exhibited no discernible impact on cell viability when administered within the range of 0–20 μM dosages (Figure 1b). Subsequently, the HepG2 cells were treated with 20 μM Lactucopicrin for 48 h. The study evaluated the impact of Lactucopicrin on FFA‐induced lipid formation in HepG2 cells using TG content and Oil red O staining. The findings revealed that Lactucopicrin significantly reduced the TG content and lipid droplet count and ameliorated FFA‐induced lipid accumulation, while FFA notably elevated the TG content and lipid droplet count and induced lipid accumulation in HepG2 cells (Figure 1c–e).

### 3.2 Lactucopicrin may promote fatty acid β oxidation to improve FFA‐induced lipid accumulation in HepG2 cells

**FIGURE 2:**
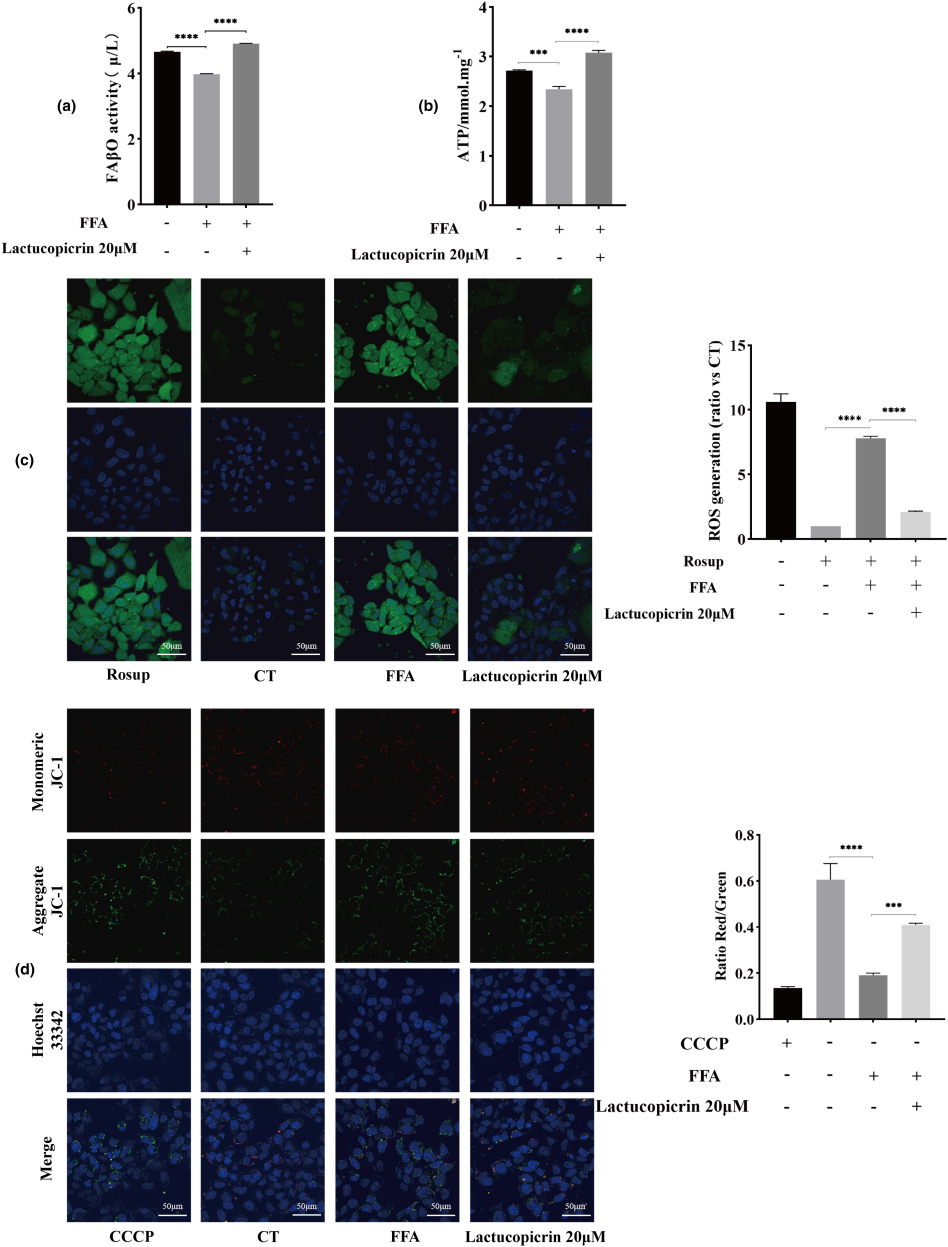
Lactucopicrin promotes fatty acid β‐oxidation to reduce FFA‐induced lipid accumulation in HepG2 cells. (a) Effect of Lactucopicrin on fatty acid β oxidase (FAβO) activity in HepG2 cells. (b) Effect of Lactucopicrin on ATP content in HepG2 cells. (c) After 48 h of drug treatment, cells were stained with dichloro‐dihydro‐fluorescein diacetate (DCFH‐DA) for 30 min. ROS relevant fluorescence amount was analyzed under confocal microscope, Scale bar = 50 μm. Fluorescence intensity of intracellular level of ROS generation is calculated and analyzed. (d) After 48 h of drug treatment, cells were stained with JC‐1 staining working solution and Hoechst 33342 for 30 min, changes in mitochondrial Δψm were determined by visualizing under confocal microscope, Scale bar = 50 μm. Intracellular level of mitochondrial membrane potential (ΔΨm) is calculated and analyzed. Data were expressed as mean ± SD for three separate experiments, ***p < .001, ^****^ *p* < .0001.

To investigate the effect of Lactucopicrin on intracellular fatty acid β‐oxidation levels, we measured fatty acid β‐oxidase (FAβO) activity, ATP content, reactive oxygen species (ROS) levels, and mitochondrial membrane potential (JC‐1). Our findings reveal that the activity of FAβO and the content of ATP in HepG2 cells were significantly reduced after FFA induction compared to the CT group. However, following the intervention of Lactucopicrin, the intracellular activity of FAβO and ATP content showed a significant increase (Figure 2a,b). The higher the level of intracellular reactive oxygen species, the stronger the intensity of green fluorescence. The intensity of green fluorescence of the cells in the FFA group increased significantly, indicating that the level of intracellular reactive oxygen species also increased significantly, and after the intervention of Lactucopicrin, the intensity of green fluorescence was reduced again, indicating that the level of intracellular reactive oxygen species decreased significantly (Figure 2c). Red fluorescence is generated at elevated levels of mitochondrial membrane potential, while green fluorescence is generated at lower levels of mitochondrial membrane potential. The alteration in mitochondrial membrane potential can be measured through the red/green fluorescence ratio. In the FFA group, the intracellular red/green fluorescence ratio was notably reduced, signifying a major decrease in mitochondrial membrane potential within the cells of the FFA group. Following supplementation with Lactucopicrin, the red/green fluorescence ratio substantially increased, indicating a marked enhancement in mitochondrial membrane potential (Figure 2d). Therefore, we speculate that Lactucopicrin decreases FFA‐induced lipid accumulation in HepG2 cells by enhancing intracellular fatty acid β‐oxidation levels.

### 3.3 Lactucopicrin ameliorates FFA‐induced lipid accumulation in HepG2 cells by promoting fatty acid β‐oxidation

**FIGURE 3:**
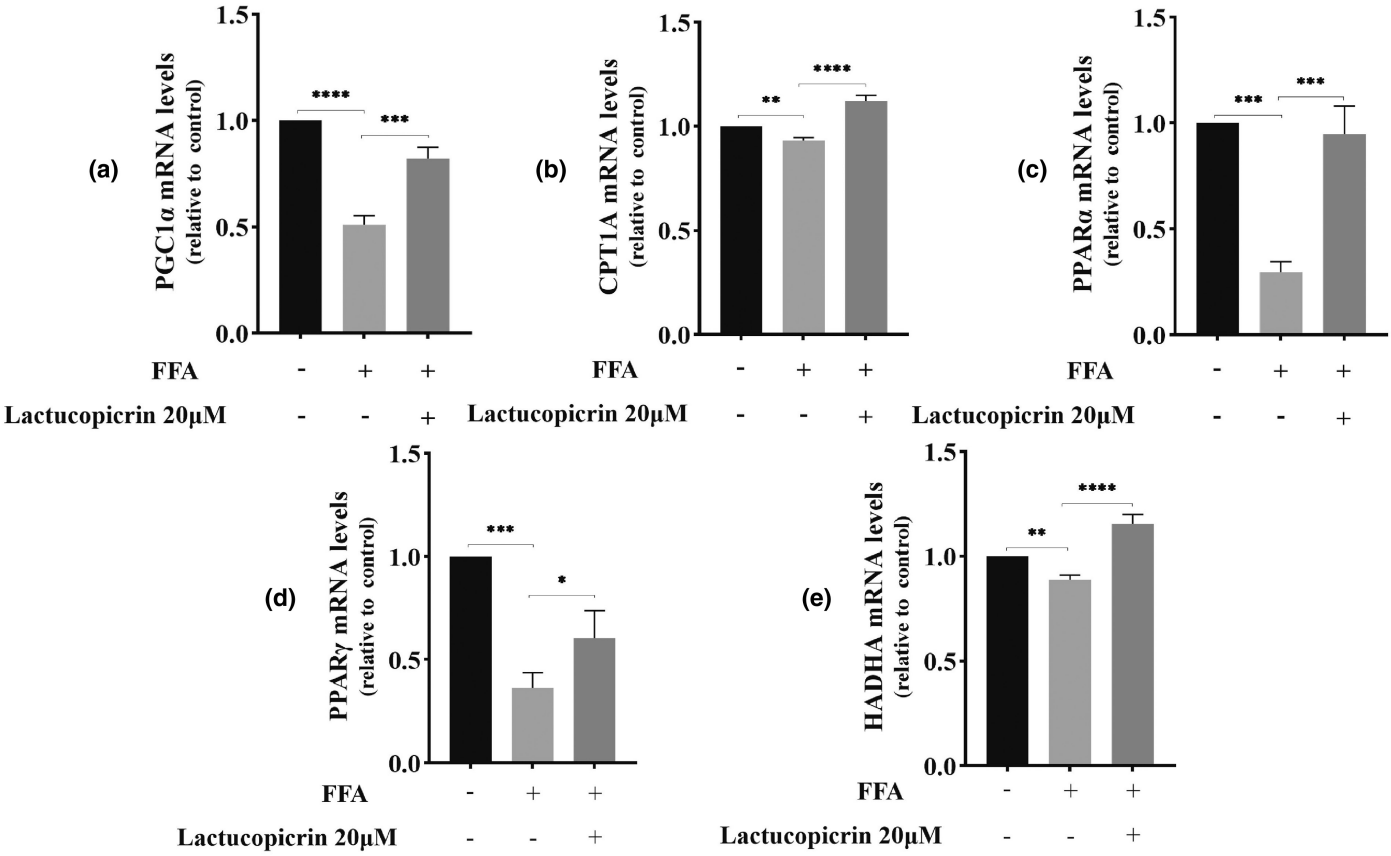
Effects of Lactucopicrin on the mRNA expression levels of fatty acid β‐oxidation factors in HepG2 cells. HepG2 cells were treated with FFA (400 μM) and/or Lactucopicrin (20 μM) for 48 h, mRNA expression levels of (a) peroxisome proliferator‐activated receptor‐γ cofactor 1α (PGC1α), (b) carnitine palmitoyltransferase 1A (CPT1A), (c) peroxisome proliferator‐activated receptor α (PPARα), (d) peroxisome proliferator‐activated receptor gamma (PPARγ), and (e) hydroxyacyl‐coenzyme A (CoA) dehydrogenase trifunctional multienzyme complex subunit alpha (HADHA) were quantified using qRT‐PCR. Data were expressed as mean ± SD for three separate experiments, *p < .05, **p < .01, ***p < .001, ^****^ *p* < .0001.

**FIGURE 4:**
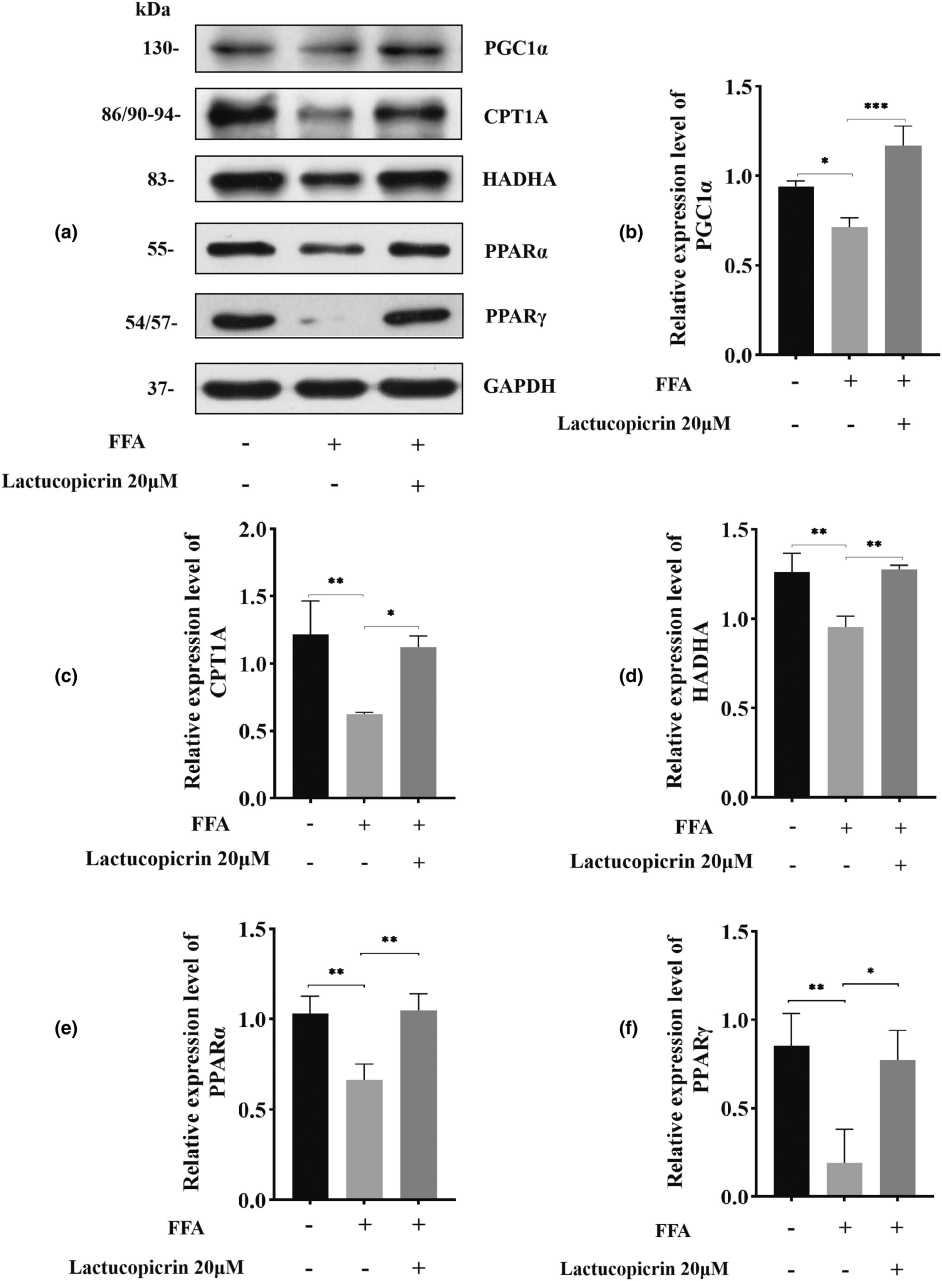
Effects of Lactucopicrin on the protein expression levels of fatty acid β‐ oxidation factors in HepG2 cells. (a) HepG2 cells were treated with FFA (400 μM) and/or Lactucopicrin (20 μM) for 48 h. Protein expression of PGC1α, CPT1A, HADHA, PPARα, and PPARγ was analyzed via Western blotting. (b–f) Densitometric analysis of the band intensity ratios of PGC1α, CPT1A, HADHA, PPARα, and PPARγ. Data were expressed as mean ± SD for three separate experiments, ^ns^ *p* > .05, *p < .05, **p < .01, ***p < .001.

The regulatory effects of Lactucopicrin on genes related to fatty acid β‐oxidation were validated using quantitative real‐time PCR. We observed a significant decrease in the relative mRNA expressions of PGC1α, PPARα, PPARγ, HADHA, and CPT1A in the FFA group compared to the CT group. Interestingly, after pretreatment with Lactucopicrin, these patterns were notably reversed (Figure 3). Furthermore, Western blot analysis confirmed the regulatory effects of Lactucopicrin on these five fatty acid β‐oxidation‐related proteins. Specifically, we noted substantial reductions in the relative protein expressions of PGC1α, HADHA, CPT1A, PPARα, and PPARγ in the FFA group compared to the control group. However, these changes can be reversed by pretreatment with Lactucopicrin (Figure 4). Based on our observations, it is conceivable that Lactucopicrin may promote the expression of PGC1α, HADHA, CPT1A, PPARα, and PPARγ, thereby enhancing fatty acid β‐oxidation.

### 3.4 Activation of AMPK pathway by Lactucopicrin and thereby promoting fatty acid β‐oxidation levels

**FIGURE 5:**
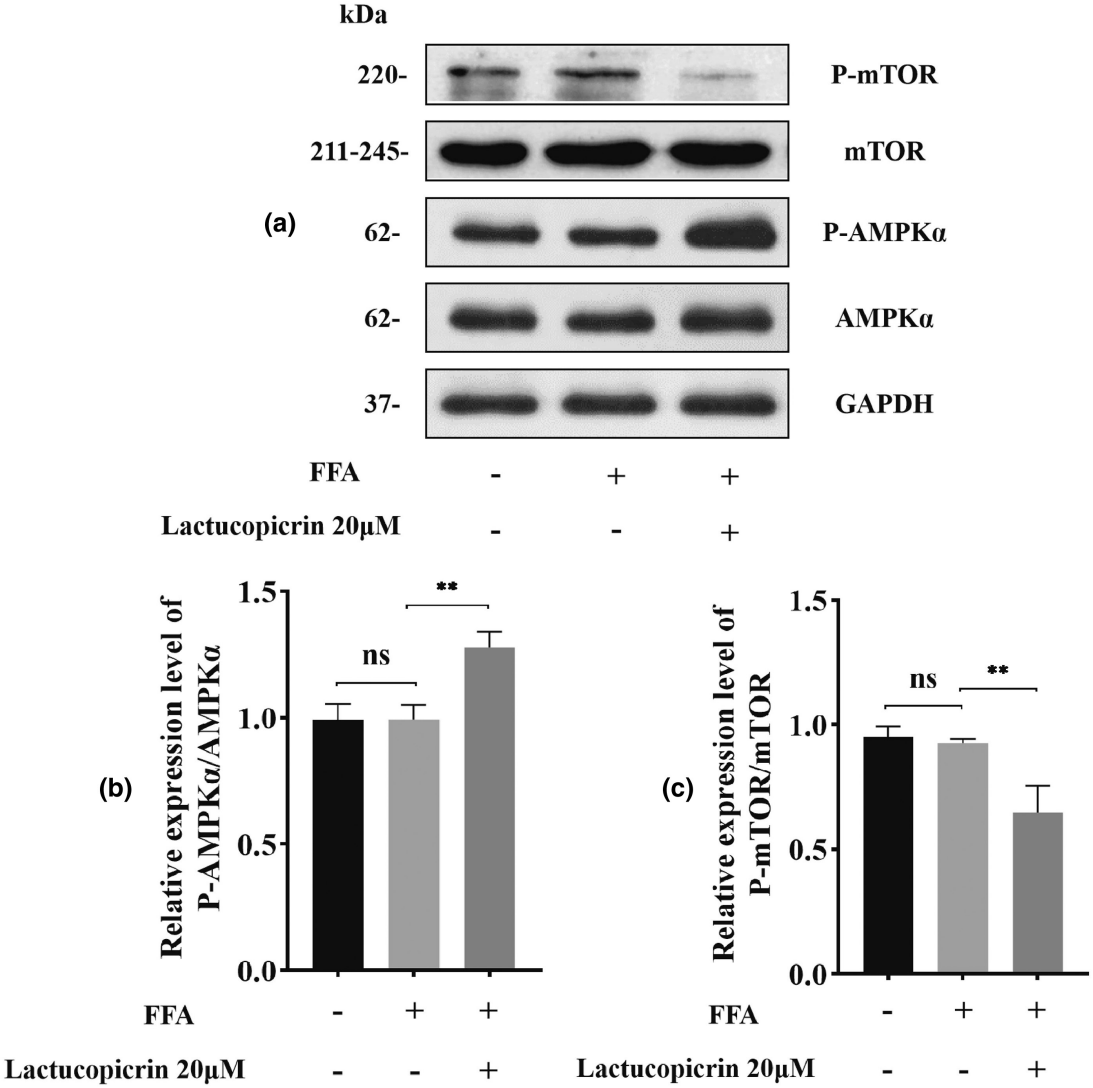
The AMPK pathway activation by Lactucopicrin treatment. (a) HepG2 cells were treated with FFA (400 μM) and/or Lactucopicrin (20 μM) for 48 h. Protein expression of AMP‐activated protein kinase α (AMPKα) and mammalian target of rapamycin (mTOR) were analyzed via Western blotting. (b, c) Densitometric analysis of the band intensity ratios of P‐mTOR/mTOR and P‐AMPKα/AMPKα. Data were expressed as mean ± SD for three separate experiments, ^ns^ *p* > .05, **p < .01.

We discovered that Lactucopicrin had almost no effect on the total protein of AMPK and mTOR, but it could significantly increase the phosphorylation level of intracellular AMPK and significantly decrease the phosphorylation level of intracellular mTOR (Figure 5). By activating the AMPK pathway, suppressing mTOR, and upregulating PGC1α, PPARα, PPARγ, CPT1A, and HADHA, Lactucopicrin enhances fatty acid β‐oxidation.

### 3.5 Dorsomorphin reverses the improvement of FFA‐induced lipid accumulation in HepG2 cells by Lactucopicrin

**FIGURE 6:**
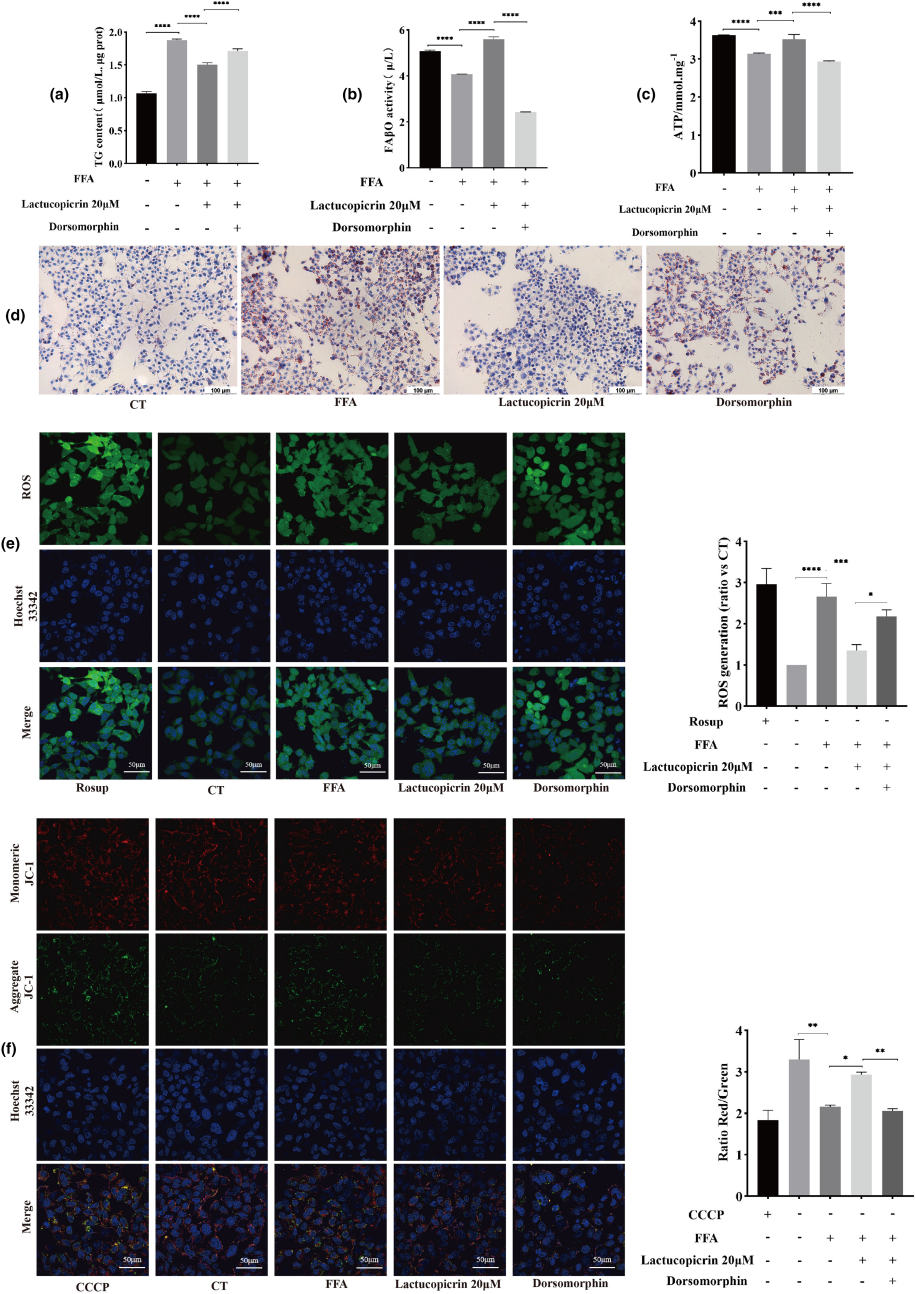
Dorsomorphin (AMPK signaling pathway inhibitor) attenuates the effect of Lactucopicrin on lipid accumulation in HepG2 cells. (a) HepG2 cells were treated with FFA (400 μM) and/or Lactucopicrin (20 μM) and/or Dorsomorphin (5 μM) for 48 h, Measurement of intracellular TG content. (b) Effect of Dorsomorphin on fatty acid β oxidase (FAβO) activity in HepG2 cells. (c) Effect of Dorsomorphin on ATP content in HepG2 cells. (d) Lipid accumulation was determined via Oil Red O staining. Scale bars, 100 μm. Control (CT), FFA 400 μM (FFA), FFA 400 μM+Lactucopicrin 20 μM (Lactucopicrin 20 μM), FFA 400 μM+Lactucopicrin 20 μM+5 μM Dorsomorphin (Dorsomorphin). (e) After 48 h of drug treatment, cells were stained with DCFH‐DA and Hoechst 33342 for 30 min. ROS relevant fluorescence amount was analyzed under confocal microscope, Scale bar = 50 μm. Fluorescence intensity of intracellular level of ROS generation is calculated and analyzed. (f) After 48 h of drug treatment, cells were stained with JC‐1 staining working solution and Hoechst 33342 for 30 min, changes in mitochondrial Δψm were determined by visualizing under confocal microscope, Scale bar = 50 μm. Intracellular level of mitochondrial membrane potential (ΔΨm) is calculated and analyzed. Data are expressed as mean ± SD for three separate experiments, *p < .05, **p < .01, ***p < .001, ^****^ *p* < .0001.

**FIGURE 7:**
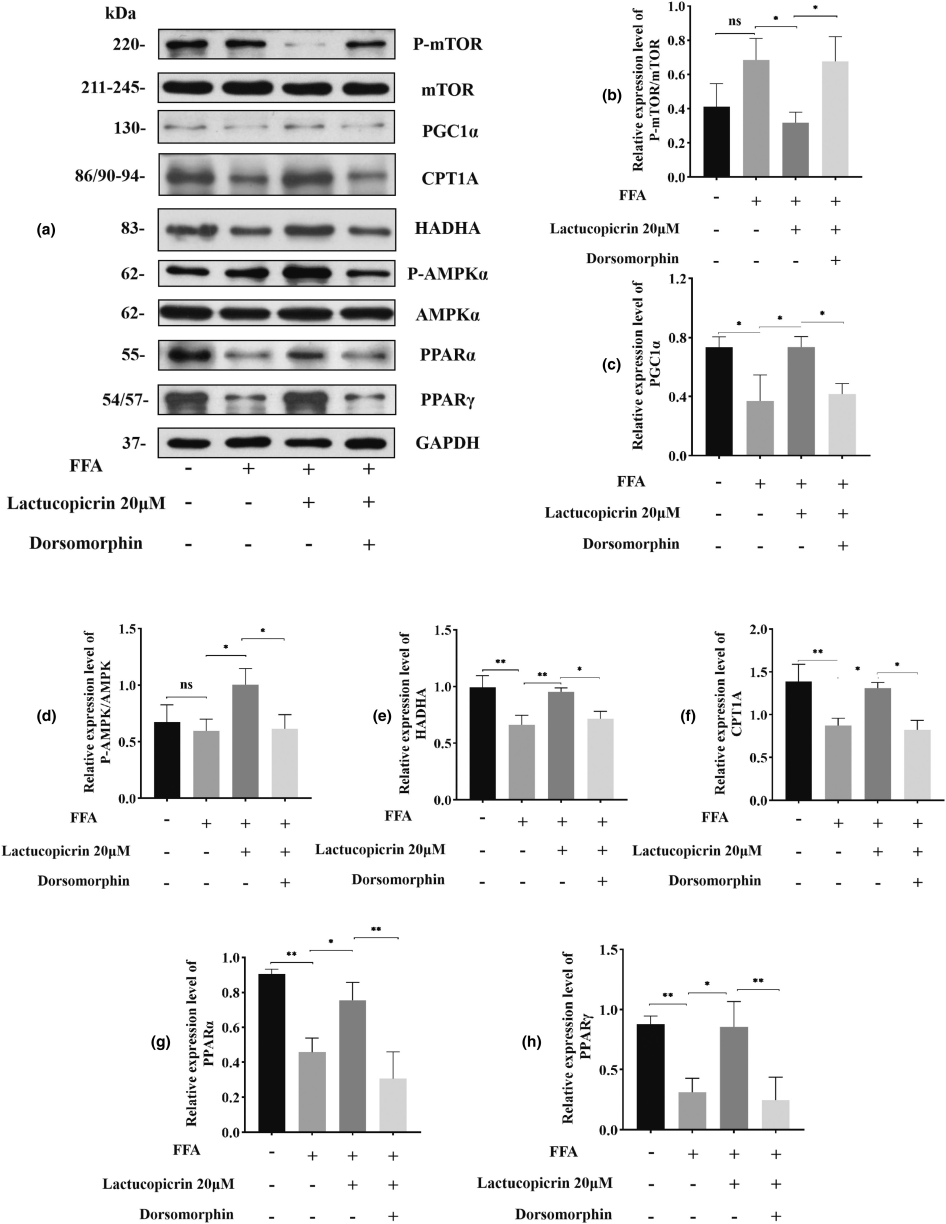
Dorsomorphin abolishes activation of AMPK and its downstream fatty acid β‐oxidation factor protein by Lactucopicrin. (a) HepG2 cells were treated with FFA (400 μM) and/or Lactucopicrin (20 μM) and/or Dorsomorphin (5 μM) for 48 h, protein expression of AMPKα, mTOR, PGC1α, CPT1A, HADHA, PPARα, and PPARγ was analyzed via Western blotting. (b–h) Densitometric analysis of the band intensity ratios of P‐mTOR/mTOR, PGC1α, CPT1A, HADHA, P‐AMPKα/AMPKα, PPARα,and PPARγ. Data are expressed as mean ± SD for three separate experiments, ns*p* > .05, *p < .05, **p < .01.

After pretreatment with Lactucopicrin, intracellular triglyceride levels decreased, while FAβO activity and ATP content increased. Pretreatment with AMPK‐specific inhibitor (Dorsomorphin) significantly reversed all of these changes (Figure 6a–c). The results of Oil red O staining, reactive oxygen species, and JC‐1 assays indicated that the effects of Lactucopicrin pretreatment, resulting in decreased lipid droplet number, decreased levels of reactive oxygen species, and increased mitochondrial membrane potential, were eliminated by Dorsomorphin pretreatment (Figure 6d–f). Dorsomorphin significantly decreased the expression of P‐AMPK/AMPK, PGC1α, PPARα, PPARγ, CPT1A, and HADHA, which were elevated by Lactucopicrin pretreatment. The P‐mTOR/mTOR ratio, decreased by Lactucopicrin pretreatment, was significantly increased upon treatment with Dorsomorphin (Figure 7). The beneficial effects of Lactucopicrin on FFA‐induced lipid accumulation in FFA‐induced HepG2 cells were significantly reversed after pretreatment with Dorsomorphin.

## 4 DISCUSSION

Lactucopicrin is a natural sesquiterpenoid widely found in plants of the Asteraceae family (chicory and lettuce) and medicinal and edible plants (Xinjiang hairy chicory). The sesquiterpene lactones in Xinjiang hairy chicory have significant hepatoprotective and hypolipidemic effects (Yang et al., 2020). First, we used the CCK8 method to determine the effect of Lactucopicrin on HepG2 cells and found that this substance had no significant effect on cell viability in the concentration range of 0–20 μM. Our previous research found that 20 μM Lactucopicrin had the best effect on lipid metabolism (Aibaidula et al., 2022), so we used this concentration to carry out the following experiments. Oleic and palmitic acids are the most abundant free fatty acids in the livers of normal individuals and patients with NAFLD, so the OA:PA = 2:1 mixture has been widely used in the cellular models of NAFLD for studying diet‐induced steatosis (Cole et al., 2018; Yazhi et al., 2011). Therefore, we established an in vitro NAFLD model with FFA‐induced HepG2 cells. Oil red O staining and triglyceride content measurement revealed the significant accumulation of cellular lipid droplets and triglycerides in the FFA group, indicating that we successfully established an in vitro NAFLD model.

We then explored the specific mechanism of Lactucopicrin in terms of fatty acid β‐oxidation. The catabolism of free fatty acids in the liver is mainly regulated by β‐oxidation, which is the core process of fatty acid catabolism (Zhang et al., 2019). One of the main mechanisms of hepatic steatosis is the damage to mitochondrial free fatty acid β‐oxidation system; the higher the activity of fatty acid β‐oxidase, the lower the risk of hepatic steatosis (da Silva Lima et al., 2022). Fatty acid β‐oxidation generally occurs in the mitochondria, which are important organelles for energy metabolism. Lipid metabolism in the liver is dependent on mitochondria, and mitochondrial fatty acid β‐oxidation is the main metabolic pathway for fatty acids in the liver (Zhou et al., 2023). Reactive oxygen species (ROS) production is one of the main features of mitochondrial dysfunction. When the ROS amount exceeds the body's ability for self‐cleaning, ROS will exacerbate hepatocellular injury, impair mitochondrial function, and ultimately lead to lipid accumulation and peroxidative damage in the liver. In turn, these changes increase ROS production and exacerbate mitochondrial damage, creating a vicious cycle (Hong et al., 2021; Rizwan et al., 2020; Tang et al., 2022). Mitochondria are also the main site of ATP production, and the ATP level can be used as an indicator of mitochondrial status and activity (Chakrabarty & Chandel, 2022). Reduced intracellular ATP content leads to slow oxidative phosphorylation, disrupting energy metabolism in hepatocytes. As a high‐energy phosphate that ensures the energy supply for all life activities in the cell, ATP can also reflect the level of energy metabolism in the cell (Lu et al., 2021). In patients with NAFLD, the activity of AMPK decreases with the ATP level in the liver (Sharma et al., 2015). The weakening of the intracellular mitochondrial membrane potential is one of the key indicators of mitochondrial dysfunction, and its stability is essential for the maintenance of normal physiological processes in the cell, mitochondrial oxidative phosphorylation, and ATP production (Luan et al., 2019). Chi Zhexu et al. also found that (Chi et al., 2020) the mitochondrial membrane potential increases with the fatty acid oxidation level. The present experimental results showed that FFAs significantly increased the intracellular ROS level and decreased the mitochondrial membrane potential and ATP content compared with those in the CT group. This finding indicated that the cellular mitochondrial function was significantly impaired in the FFA group, leading to an impaired fatty acid oxidation function. After the intervention of Lactucopicrin, the intracellular ROS level was significantly reduced compared with that of the FFA group. In addition, the mitochondrial membrane potential and ATP content were significantly increased, leading to a significant decrease in the number of intracellular lipid droplets and the content of triglyceride. This result revealed that Lactucopicrin has a certain degree of protective effect on mitochondria, and such mechanism also promotes fatty acid β‐oxidation and inhibits intracellular lipid accumulation.

Fatty acid oxidation also involves several key enzymes and key factors. As an important protein of the mitochondrial outer membrane, CPT1A facilitates the entry of fatty acids into the mitochondria to participate in fatty acid oxidation. CPT1A is a key enzyme in fatty acid β‐oxidation in the mitochondria and serves as a key indicator for evaluating this function (Moody et al., 2019). HADHA acts as a mitochondrial peroxidase that promotes fatty acid metabolism by catalyzing the last three steps of the mitochondrial β‐oxidation of long‐chain fatty acids; decreased HADHA expression results in impaired fatty acid oxidation, leading to a variety of metabolic diseases (Ding et al., 2023; Pan et al., 2022). The transcription factor PPAR is also a novel target for the treatment of liver disease and synergizes with PGC1α to promote CPT1A expression, which in turn increases fatty acid β‐oxidation; their cooperation plays a key role in fatty acid β‐oxidation (Sun et al., 2018). PPARγ is also an important regulator of lipid metabolism, and clinical studies demonstrated that PPARγ agonists can significantly reduce hepatic steatosis and improve NAFLD symptoms (Skat‐Rørdam et al., 2019). Multiple studies demonstrated that PPARγ shifts far away from liver tissues by regulating downstream genes, reducing triglyceride accumulation in cells, and increasing cholesterol efflux, resulting in the inhibition of hepatic tissue steatosis and amelioration of hepatocellular injury (Li et al., 2021; Ma et al., 2022; Wei & Huang, 2019). Meanwhile, the coactivator PGC1α synergistically activates the transcription factor PPAR, acts as a key factor in mitochondrial biosynthesis, and regulates fatty acid β‐oxidation (Rupasinghe et al., 2016). Our experimental results showed that Lactucopicrin significantly activated coactivators (PGC1α), transcription factors (PPARα and PPARγ), and oxidizing factors (HADHA and CPT1A) at the mRNA and protein levels. Therefore, we hypothesized that Lactucopicrin acts on the above key targets to promote fatty acid β‐oxidation and regulate hepatic lipids.

Adenosine monophosphate‐activated protein kinase (AMPK) and mTOR are closely linked to PGC1α and thus attracted our interest. AMPK is a central regulator of a variety of metabolic pathways, and its role in glycolipid metabolism rendered it a potential molecular target for NAFLD (Lee et al., 2018). Multiple studies showed that the activation of the AMPK pathway can effectively improve high‐fat diet‐induced NAFLD (Shen et al., 2023; Zou et al., 2021). mTOR is the opposite of AMPK. As described by Asier González (González et al., 2020), AMPK and mTOR can be regarded as yin and yang, sensing different nutrient states, regulating opposite metabolic processes, and exercising their corresponding biological functions. Researchers at the University of Pennsylvania also showed that the selective inhibition of the mTOR pathway benefits hepatic lipid metabolism and prevents the onset and progression of NAFLD (Gosis et al., 2022). In our study, Lactucopicrin significantly activated AMPK and inhibited mTOR compared with those in the FFA group. We hypothesized that Lactucopicrin might be a potential agonist of AMPK. To confirm this idea, we added AMPK‐specific inhibitor (Dorsomorphin) for further verification. The results showed that Lactucopicrin activated the coactivator PGC1α by triggering the AMPK pathway and inhibiting mTOR. Ying Guo's findings (Guo et al., 2023) were similar: when activated, AMPK can directly trigger PGC1α while inhibiting mTOR to maintain normal mitochondrial function. Our study also revealed that PGC1α activation regulated the transcription factors PPARα and PPARγ involved in fatty acid metabolism. The enhancement of the transcription factors also significantly increased the regulation of downstream target proteins. The activation of these important targets involved in fatty acid oxidation maintains the normal function of the mitochondria and effectively inhibits lipid accumulation, which is important in lipid metabolism.

Lactucopicrin is a bioactive ingredient extracted from Xinjiang hairy chicory. In this study, we established an in vitro NAFLD model to investigate the interventional role of Lactucopicrin in lipid metabolism. Results showed that Lactucopicrin promotes fatty acid β‐oxidation, reduces intracellular lipid accumulation, and inhibits NAFLD development by activating the AMPK pathway and its downstream‐related factors, which are the key targets of NAFLD. These result suggest that the multitarget agonism of Lactucopicrin makes it a potential candidate for NAFLD treatment.

## AUTHOR CONTRIBUTIONS

**Huiwen Tan:** Data curation (equal); validation (lead); writing – original draft (lead). **Na Mi:** Data curation (equal); writing – original draft (supporting). **Fenglian Tong:** Investigation (equal); writing – review and editing (equal). **Rui Zhang:** Investigation (equal); writing – review and editing (equal). **Adalaiti Abudurexiti:** Methodology (lead); supervision (supporting). **Yi Lei:** Data curation (supporting); validation (supporting). **Yewei Zhong:** Formal analysis (equal); methodology (supporting). **Junlin Yan:** Formal analysis (equal); methodology (supporting). **Jian Yang:** Resources (supporting). **Xiaoli Ma:** Funding acquisition (lead); resources (lead); supervision (lead).

## FUNDING INFORMATION

This research was supported by the Xinjiang Uygur Autonomous Region Young Top Talent Program (Grant No. 2022TSYCCX0104).

## CONFLICT OF INTEREST STATEMENT

All authors certify that they have participated sufficiently in the work to take public responsibility for the appropriateness of the experimental design and method, and the collection, analysis, and interpretation of the data. The authors have reviewed the final version of the manuscript and approved it for publication. To the best of our knowledge and belief, this manuscript has not been published in whole or in part nor is it being considered for publication elsewhere.

## Data Availability

The data presented in the manuscript are available on request from the corresponding author.
